# Phase 2 study of everolimus for relapsed or refractory classical Hodgkin lymphoma

**DOI:** 10.1186/s40164-018-0103-z

**Published:** 2018-05-11

**Authors:** Patrick B. Johnston, Lauren C. Pinter-Brown, Ghulam Warsi, Kristen White, Radhakrishnan Ramchandren

**Affiliations:** 1 Division of Hematology, Mayo Clinic, 200 First Street SW, Rochester, MN 55905 USA; 2 Department of Hematology/Oncology, David Geffen School of Medicine at UCLA, Los Angeles, CA USA; 3 Department of Oncology, Novartis Pharmaceuticals Corporation, East Hanover, NJ USA; 4 Department of Hematology/Oncology, Karmanos Cancer Institute, Detroit, MI USA

**Keywords:** Clinical trial, Everolimus, Hodgkin lymphoma, mTOR inhibitors, Relapsed/refractory

## Abstract

**Background:**

The current standard of care for classical Hodgkin lymphoma (HL) is multiagent chemotherapy with or without radiation. In patients who relapse or fail to respond, additional high-dose chemotherapy with autologous hematopoietic stem cell transplantation (AHSCT) can improve progression-free survival (PFS). Novel therapies are required for patients refractory to chemotherapy and AHSCT. The mammalian target of rapamycin inhibitor everolimus has shown preliminary activity in preclinical models of HL and promising efficacy in patients with relapsed or refractory HL.

**Methods:**

This was an open-label, two-stage, phase 2 study that enrolled 57 patients aged ≥ 18 years with classic HL that had progressed after standard therapy. Patients received everolimus 10 mg daily until disease progression, intolerable toxicity, withdrawal of consent, or investigator decision. The primary endpoint was overall response rate; secondary endpoints included PFS, overall survival, time to response, duration of response, and safety.

**Results:**

Overall response rate was 45.6% (95% confidence interval [CI] 32.4–59.3%); five patients (8.8%) experienced a complete response and 21 patients had a partial response (36.8%). Median PFS was 8.0 months (95% CI 5.1–11.0 months). Seven patients (12%) were long-term responders (≥ 12 months). The most common study drug-related adverse events were thrombocytopenia (45.6%), fatigue (31.6%), anemia (26.3%), rash (24.6%), and stomatitis (22.8%).

**Conclusions:**

Everolimus 10 mg/day demonstrated favorable results in patients with heavily pretreated, relapsed, or refractory classical HL. These findings support the further evaluation of everolimus in this indication.

*Trial registration* ClinicalTrials.gov NCT01022996. Registered November 25, 2009

## Background

In 2014 there were more than 9000 estimated new cases of Hodgkin lymphoma (HL) in the United States, and an estimated 1180 deaths were caused by the disease [1]. The highest incidence of new HL diagnoses occurred in patients between the ages of 20 and 34 years [1, 2]. The 5-year relative survival rate improved from approximately 70% in 1975 to just over 88% in 2006 [1]. The current standard of care for classical HL is systemic chemotherapy, with the main options being ABVD [Adriamycin^®^ (doxorubicin), bleomycin, vinblastine, and dacarbazine], Stanford V (doxorubicin, vinblastine, mechlorethamine, etoposide, vincristine, bleomycin, and prednisone), or BEACOPP (bleomycin, etoposide, doxorubicin, cyclophosphamide, oncovin (vincristine), procarbazine, and prednisone), with or without the addition of radiotherapy [3]. For patients who do not respond to first-line treatment (≈ 10%) or who relapse after experiencing initial response (≈ 20%), high-dose chemotherapy with autologous hematopoietic stem cell transplantation (AHSCT) is the standard of care [3–6]. This salvage therapy has been shown to improve progression-free survival (PFS) and event-free survival [4, 7–9].

Patients whose disease is refractory to or relapses after high-dose chemotherapy with AHSCT have traditionally had limited treatment options, although a growing understanding of the biology of HL is leading to the development and testing of a wider range of novel therapeutic strategies [6, 10]. Allogeneic hematopoietic stem cell transplantation (allogeneic HSCT) has been associated with very high treatment-related mortality rates and limited success, although there are case histories reporting long-term remission using this strategy [11]. Less toxic post-AHSCT therapies are urgently needed. The anti-CD30 antibody-drug conjugate brentuximab vedotin, which has shown benefit in patients with relapsed or refractory HL [12, 13], has been approved in the United States and Europe for the treatment of HL after failure of AHSCT or after failure of two or more previous multiagent chemotherapy regimens in patients who are not candidates for AHSCT [3]. In a phase 2 study in 102 patients with relapsed/refractory HL, the overall response rate (ORR) for brentuximab vedotin was 75%, with a disease control rate (DCR) of 96 and 34% achieving complete response (CR) [13]. In patients with a CR, the median PFS was 21.7 months [13]. Despite these encouraging results, the overall median PFS was only 5.6 months across the trial’s entire patient population [13]. Moreover, as brentuximab vedotin is not considered to be curative therapy, all patients are expected to eventually relapse. More recently, immune checkpoint inhibitors (CPIs) targeting the programmed death pathway have been developed to reinstate anti-tumor immune responses [14], and two such agents (nivolumab and pembrolizumab) are now approved for the treatment of refractory HL [3, 15]. Nivolumab is a treatment option for relapsed/refractory HL following HSCT, and pembrolizumab is an option for relapsed/refractory HL after ≥ 3 prior lines of therapy [3]. However, accelerated approval granted for both of these agents was based on overall response rate and duration of response, and confirmatory data of their clinical benefit in refractory HL may be required [16, 17]. Therefore, there remains an unmet need for additional therapeutic options to improve outcomes for more of these heavily pretreated patients.

The phosphatidylinositol 3-kinase (PI3K)/Akt/mammalian target of rapamycin (mTOR) pathway, a central regulator of cell growth, proliferation, survival, metabolism, and angiogenesis, is often dysregulated in HL [18–21]. Everolimus is an oral mTOR inhibitor that has demonstrated antitumor activity in refractory hematologic malignancies [22, 23] and in preclinical studies in both in vitro and in vivo models of HL [24]. In a phase 2 trial of inpatients with relapsed or refractory lymphomas treated with everolimus 10 mg once daily, a subgroup of 19 patients with HL responded positively to treatment with everolimus, with an ORR of 47% (9/19), a median duration of response of 7.1 months, and median PFS and overall survival (OS) of 6.2 and 25.2 months, respectively [25]. Treatment was generally well tolerated; the most common grade 3/4 adverse events (AEs) possibly related to everolimus were anemia (32%), thrombocytopenia (32%), dyspnea (10%), and pneumonia (10%) [25]. Following these promising results, the current prospective phase 2 trial was undertaken to confirm the efficacy and safety of everolimus in patients with relapsed or refractory HL.

## Methods

### Patients

The study enrolled patients aged ≥ 18 years with classical HL that had progressed after high-dose chemotherapy with AHSCT (if eligible) and/or a gemcitabine-, vinorelbine-, or vinblastine-containing regimen. Patients were required to have an Eastern Cooperative Oncology Group performance status of ≤ 2, at least one site of measurable disease, and adequate bone marrow, hepatic, and renal function. They also had to meet the following blood and chemistry measures: absolute neutrophil count ≥ 1.0 × 10^9^/L, platelet count ≥ 75 × 10^9^/L, serum creatinine ≤ 1.5 × upper limit of normal (ULN), serum bilirubin ≤ 1.5 × ULN, aspartate and alanine aminotransferase levels ≤ 2.5 × ULN or ≤ 5.0 × ULN if elevation is due to disease involvement, fasting cholesterol ≤ 300 mg/dL (≤ 7.75 mmol/L), and fasting triglycerides ≤ 2.5 × ULN. No concomitant anticancer therapy was permitted. Patients were ineligible for the trial if they had received any prior treatment with an mTOR inhibitor; if they were treated with a monoclonal antibody therapy, chemotherapy, any investigational drug, or radiation therapy, or if they had had major surgery within 4 weeks of starting everolimus. Patients were also not permitted to have had previous allogeneic HSCT nor any other severe or uncontrolled medical conditions.

### Study design and treatment

This was a multicenter, open-label, single-arm, two-stage, phase 2 study conducted in the United States (ClinicalTrials.gov identifier NCT01022996, https://clinicaltrials.gov/ct2/show/NCT01022996). Everolimus was administered orally at a dose of 10 mg once daily (at the same time every day, with or without food) until disease progression, intolerable toxicity, withdrawal of consent, or investigator decision. Dose adjustments and interruptions were permitted according to an algorithm outlined in the study protocol in the event of toxicity suspected to be related to everolimus.

All patients (or their legal representative) provided written informed consent before enrollment. The study was conducted in accordance with the ICH Good Clinical Practice Guidelines and the ethical principles specified in the Declaration of Helsinki. The protocol and consent forms were reviewed and approved by the appropriate ethics body of each institution before study initiation.

### Efficacy and safety evaluations

Tumor assessments were performed at baseline using integrated positron emission tomography (PET)/computed tomography (CT) with contrast and every 12 weeks (3 cycles) thereafter using integrated PET/CT with contrast or CT with intravenous contrast. In the event of CR or partial response (PR), respectively, a confirmatory scan was performed 4 or more weeks after the response was first documented.

The primary study endpoint was ORR. Secondary endpoints included DCR, PFS, OS, time to overall response, duration of overall response, and duration of disease control. Safety was assessed by monitoring and recording all AEs and regularly monitoring hematology, serum chemistry, and physical condition. Toxicity was assessed according to the National Cancer Institute Common Terminology Criteria for Adverse Events, version 3.0.

### Statistics

The trial used a Simon two-stage MinMax design [26]. The trial required enrollment of 54 eligible patients to test the null hypothesis that the true ORR for everolimus is ≤ 15% versus the alternative hypothesis that the true ORR is ≥ 30%. The study had 85% power with a one-sided type I error rate of 5%. Stage 1 was to enroll 39 patients; if ≤ 7 confirmed responses were noted, the trial was to be discontinued. If ≥ 8 confirmed responses were observed among the first 39 patients, an additional 15 patients were to be enrolled in stage 2.

Efficacy analyses were performed using the full analysis set of patients, defined as all patients who received at least one dose of study drug. The safety assessment was performed on the safety population, defined as all enrolled patients who received at least one dose of study drug and had at least one postbaseline safety assessment.

Exact 95% confidence intervals (CIs) were calculated for ORR and DCR. Progression-free survival, OS, duration of response, and duration of disease control were estimated using the Kaplan–Meier method. ORR was defined as the percentage of patients who achieved confirmed CR or PR within 32 weeks of everolimus initiation as determined by CT scan using modified response criteria for malignant lymphoma, adapted from Cheson et al. [27]. PFS was defined as the time from start of treatment to the date of documented disease progression, death from any cause, or start of new anticancer therapy. OS was defined as the time from start of treatment to the date of death from any cause. DCR was defined as the percentage of patients who achieved a best overall response of CR, PR, or stable disease, and duration of disease control was defined as the time from start of treatment to the date of documented disease progression, death from any cause, or start of new anticancer therapy in patients who experienced CR, PR, or stable disease. Time to overall response was measured as the time from the start of treatment to the first documented response, and duration of overall response was defined as the time from the first documented response to the date of documented disease progression, death from any cause, or start of new anticancer therapy. Patients who were still receiving treatment at the time of the data analysis were censored at the date of the last adequate tumor assessment.

## Results

### Patient characteristics

A total of 57 patients from 14 centers were enrolled between December 22, 2009, and November 24, 2014. The study ran until November 28, 2014, when the last patient discontinued (i.e., completed the study). Reasons for treatment end were disease progression (*n* = 32), AEs (*n* = 14), withdrawal of consent (*n* = 3), administrative problems (*n* = 6), and protocol deviation or lost to follow-up (*n* = 1 each). Eleven patients completed the study follow-up phase according to protocol.

Patient demographics and baseline disease characteristics are summarized in Table 1. The median patient age was 32.0 years, 58% of patients were female, and 77% were white. Previous therapy included a gemcitabine-, vinorelbine-, or vinblastine-containing regimen in 100% of patients and AHSCT in 67% of patients. In addition, 15 patients (26.3%) received prior brentuximab vedotin, with CR and PR being achieved by three patients each (duration of response ranging from 1 to 11 months). Two-thirds of patients had experienced disease progression during previous therapy.

**Table 1:** Baseline demographics and disease characteristics

Characteristic, n (%)	*N* = 57
Age, years, median (range)	32.0 (19.0–77.0)
Sex, n (%)	
Male	24 (42.1)
Female	33 (57.9)
Race, n (%)	
White	44 (77.2)
Black or African American	8 (14.0)
Asian	1 (1.8)
Pacific Islander	3 (5.3)
Other	1 (1.8)
ECOG performance status, n (%)	
0	32 (56.1)
1	23 (40.4)
2	2 (3.5)
Hodgkin lymphoma classification, n (%)	
Classical nodular sclerosis	52 (91.2)
Classical mixed cellularity	3 (5.3)
Other	2 (3.5)
Stage at diagnosis, n (%)	
I	2 (3.5)
II	20 (35.1)
III	18 (31.6)
IV	17 (29.8)
Number of previous regimens, median (range)	4 (1–17)
Previous treatment, n (%)	
AHSCT	38 (66.7)
Gemcitabine-containing regimen	32 (56.1)
Vinorelbine-containing regimen	23 (40.4)
Vinblastine-containing regimen	55 (96.5)
Disease progression during previous therapy, n (%)	38 (66.7)
Time from diagnosis to first recurrence/relapse, months, median (range)	11.1 (0.6–80.0)
Time from diagnosis to most recent recurrence/relapse, months, median (range)	38.9 (7.0–221.4)

Unless otherwise noted, all data are presented as n (%)

*AHSCT* autologous hematopoietic stem cell transplantation, *ECOG* Eastern Cooperative Oncology Group

### Efficacy

**Fig. 1:**
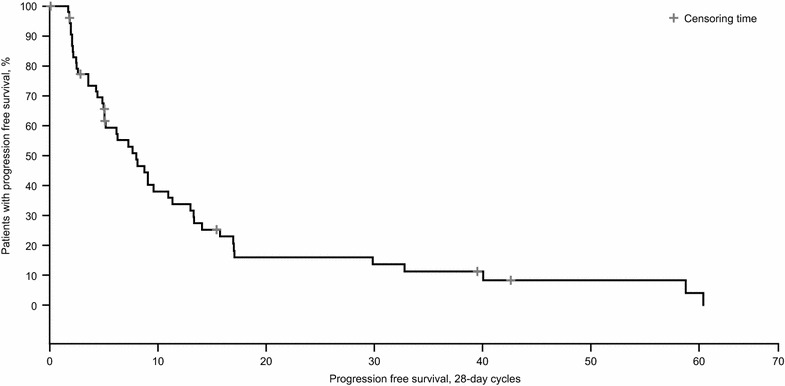
Kaplan–Meier estimate of progression-free survival

**Fig. 2:**
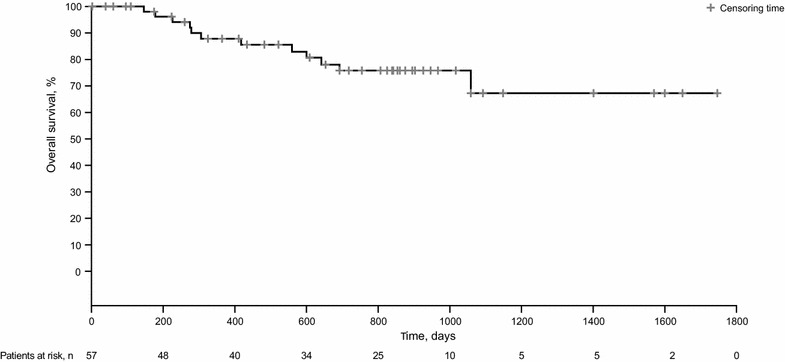
Kaplan–Meier estimate of overall survival

The ORR was 45.6% (*n* = 26/57; 95% CI 32.4–59.3%); five patients experienced a CR (8.8%) and 21 experienced a PR (36.8%) (Table 2). An additional 20 patients (35.1%) experienced disease stabilization, indicating a DCR of 80.7%. Median duration of response was 7.3 months (range 1 day–4.2 years). Median PFS was 7.3 months (95% CI 4.7–10.1 months) (Fig. 1). Median OS was not reached (Fig. 2); 45 patients were still alive (78.9%) at the time of analysis.

**Table 2:** Best overall response to everolimus treatment

Best overall response, n (%)	*N* = 57
Complete response	5 (8.8)
Partial response	21 (36.8)
Stable disease	20 (35.1)
Progressive disease	9 (15.8)
Unknown	2 (3.5)

Seven patients had a response duration of ≥ 1 year (i.e., long-term responders) (Table 3). Best response for these patients included two CRs and five PRs. Two patients experienced prolonged stable disease prior to response (1.8 years before CR and 1.2 years before PR, respectively), and one patient had a prolonged PR followed by CR (over 3 years in total). One patient with a PR has been on everolimus therapy for 4.7 years.

**Table 3:** Detailed information on long-term responders to everolimus

Patient	Previous therapy		Everolimus			
	Systemic therapy (best response)	Other	Treatment duration, years	Best response	Current response (date)	Response duration, years
White male, aged 64 years	1. ABVD (UNK) 2. ICE (UNK)	Radiotherapy ASCT	4.7	PR	PD (August 8, 2014)	4.2
White female, aged 28 years	1. ABVD (PR) 2. ICE (PR) 3. DHAP (PR)	Radiotherapy ASCT	3.9	PR	PD (October 28, 2014)	3.5
Asian female, aged 28 years	1. ABVD (PR) 2. ICE (PR)	Radiotherapy	2.5	PR	PD (November 5, 2013)	2.2
White female, aged 36 years	1. ABVD (PR) 2. ICE (NA) 3. CBV (NA) 4. Panobinostat (stable disease)	Radiotherapy ASCT (× 2)	4.5	CR	PD (November 24, 2014)	2.3^a^
White male, aged 39 years	1. ABVD (CR) 2. ICE (CR)	Radiotherapy ASCT	3.8	CR	Unknown (October 8, 2014)	3.7^b^
White male, aged 40 years	1. ABVD (PR) 2. ICE (NA) 3. GVD (NA) 4. SWOG protocol 14110 (CR) 5. Brentuximab (stable disease) 6. HDAC inhibitor (UNK)	Radiotherapy ASCT (× 2)	3.1	PR	PD (May 27, 2014)	1.8^a^
White female, aged 27 years	1. ABVD (PR) 2. ICE (PR) 3. Mini-BEAM (NA) 4. Brentuximab (stable disease) 5. Lenalidomide (stable disease)	Radiotherapy ASCT	4.0	PR	Unknown (September 4, 2014)	1.0

*ABVD* Adriamycin (doxorubicin), bleomycin, vinblastine, and dacarbazine, *AHSCT* autologous hematopoietic stem cell transplantation, *CBV* cyclophosphamide, carmustine, etoposide, *CR* complete response, *DHAP* rituximab, dexamethasone, cytarabine, cisplatin, *GVD* gemcitabine, vinorelbine, doxil, *HDAC* histone deacetylase, *ICE* ifosfamide, carboplatin, etoposide, *mini-BEAM* carmustine, etoposide, cytarabine, melphalan, *PD* progressive disease, *PR* partial response, *SWOG* southwest oncology group, *UNK* unknown

^a^ Prior to response, patient experienced prolonged stable disease

^b^ Duration of both CR and PR

### Safety

Three patients died from their cancer during the study. The median duration of exposure to everolimus was 4.1 months (range 1 day–4.7 years). Nearly a quarter of patients (22.8%) received everolimus for more than 13 months. Dose reductions were required in 35 patients (61.4%), and 31 patients (54.4%) experienced dose interruptions. AEs, which were the most common reason for dose adjustment, were experienced by 30 patients (52.6%); laboratory test abnormalities led to dose adjustments in an additional 14% of patients.

All but one patient experienced at least one AE of any grade during the study (Table 4). Grade 3/4 AEs occurred in 35 patients (61.4%). The most common AEs were fatigue (57.9%), thrombocytopenia (49.1%), and cough (49.1%); most were grade 1/2 in severity (100, 57.1, and 96.4%, respectively). Stomatitis was experienced by 24.6% of patients, but was of grade 3/4 in only 3.5% of patients. Pneumonitis was observed in six patients (10.5%) and was of grade 1 in one patient (1.8%) and grade 2 in five patients (8.8%); no patients experienced grade 3/4 pneumonitis. No grade 4 hyperglycemia, hypercholesterolemia, or hypertriglyceridemia events were observed. Serious AEs were experienced by 18 (31.6%) patients. Serious AEs occurring in more than one patient included pneumonia in four patients (grade 2, *n* = 1; grade 3, *n* = 3), pyrexia in three patients (grade 1, *n* = 1; grade 2, *n* = 2), anemia in two patients (grade 1, *n* = 1; grade 4, *n* = 1), pleural effusion in two patients (grade 2, *n* = 1; grade 3, *n* = 1), tachycardia in two patients (grade 1, *n* = 1; grade 2, *n* = 1), and hypotension in two patients (grade 1, *n* = 1; grade 2, *n* = 1).

**Table 4:** Adverse events of any cause that were experienced by > 10% of patients (*N* = 57)

AE	Any grade	Grade 1/2	Grade 3/4
Any	56 (98.2)	21 (36.8)	35 (61.4)
Fatigue	33 (57.9)	33 (57.9)	0
Thrombocytopenia	28 (49.1)	16 (28.1)	12 (21.1)
Cough	28 (49.1)	27 (47.4)	1 (1.8)
Rash	22 (38.6)	21 (36.8)	1 (1.8)
Anemia	19 (33.3)	11 (19.3)	8 (14.0)
Pyrexia	19 (33.3)	19 (33.3)	0
Dyspnea	17 (29.8)	14 (24.6)	3 (5.3)
Back pain	16 (28.1)	14 (24.6)	2 (3.5)
Diarrhea	16 (28.1)	15 (26.3)	1 (1.8)
Stomatitis	14 (24.6)	12 (21.1)	2 (3.5)
Upper respiratory tract infection	14 (24.6)	13 (22.8)	1 (1.8)
Headache	13 (22.8)	12 (21.1)	1 (1.8)
Nausea	14 (24.6)	14 (24.6)	0
Vomiting	13 (22.8)	13 (22.8)	0
Peripheral edema	12 (21.1)	12 (21.1)	0
Hyperglycemia	10 (17.5)	7 (12.3)	3 (5.3)
Pruritus	12 (21.1)	12 (21.1)	0
Abdominal pain	9 (15.8)	9 (15.8)	0
Arthralgia	9 (15.8)	9 (15.8)	0
Aspartate aminotransferase increased	9 (15.8)	8 (14.0)	1 (1.8)
Muscle spasms	9 (15.8)	9 (15.8)	0
Neuropathy peripheral	9 (15.8)	9 (15.8)	0
Oropharyngeal pain	11 (19.3)	11 (19.3)	0
Acne	8 (14.0)	8 (14.0)	0
Alanine aminotransferase increased	8 (14.0)	7 (12.3)	1 (1.8)
Dysgeusia	8 (14.0)	8 (14.0)	0
Epistaxis	8 (14.0)	8 (14.0)	0
Neutropenia	8 (14.0)	3 (5.3)	5 (8.8)
Pain	8 (14.0)	7 (12.3)	1 (1.8)
Pain in extremity	8 (14.0)	7 (12.3)	1 (1.8)
Sinusitis	10 (17.5)	10 (17.5)	0
Blood alkaline phosphatase increased	9 (15.8)	7 (12.3)	2 (3.5)
Bronchitis	7 (12.3)	7 (12.3)	0
Hypertriglyceridemia	7 (12.3)	5 (8.8)	2 (3.5)
Pneumonia	7 (12.3)	3 (5.3)	4 (7.0)
Blood lactate dehydrogenase increased	6 (10.5)	6 (10.5)	0
Decreased appetite	7 (12.3)	7 (12.3)	0
Hypercholesterolemia	6 (10.5)	6 (10.5)	0
Hypokalemia	6 (10.5)	5 (8.8)	1 (1.8)
Hypophosphatemia	7 (12.3)	2 (3.5)	5 (8.8)
Insomnia	6 (10.5)	6 (10.5)	0
Night sweats	6 (10.5)	6 (10.5)	0
Pleural effusion	6 (10.5)	4 (7.0)	2 (3.5)
Pneumonitis	6 (10.5)	6 (10.5)	0
Weight decreased	7 (12.3)	7 (12.3)	0

*AE* adverse event

The most common AEs (> 20%) considered to be related to study drug administration were thrombocytopenia (45.6% overall; 21.1% grade 3/4), fatigue (31.6% overall; 0% grade 3/4), anemia (26.3% overall; 12.3% grade 3/4), rash (24.6% overall; 1.8% grade 3/4), and stomatitis (22.8% overall; 3.5% grade 3/4). AEs that led to permanent study drug discontinuation in more than one patient included thrombocytopenia in four patients (7.0%) and dyspnea and pyrexia in two patients (3.5%) each. Overall, 54.4% of patients had an AE that required study drug dose adjustment or interruption; the most common AEs were thrombocytopenia (19.3%), neutropenia (8.8%), anemia (7.0%), and hypophosphatemia (7.0%). Only one case (1.8%) of grade 2 pneumonitis required adjustment or interruption of study drug dose.

## Discussion

Everolimus 10 mg once daily provided a favorable ORR of 45.6% and a median PFS of 7.3 months in patients with heavily pretreated, relapsed, or refractory classical HL. The ORR of nearly 46% is similar to the 47% reported for patients with HL in a phase 2 study of everolimus monotherapy in rare lymphomas [25]. The DCR of 80.7% is a positive finding in this heavily pretreated patient population, which had a median of four previous treatments. Despite the ORR in this study being considerably lower than that reported for brentuximab vedotin in a single-arm phase 2 trial (ORR 75%), the PFS was encouragingly longer with everolimus (8.0 months) than the 5.6 months reported for brentuximab in patients with CD30+ relapsed or refractory HL following high-dose chemotherapy with AHSCT [13]. A pivotal phase 2 study in heavily pretreated patients receiving brentuximab vedotin also reported a substantially higher ORR (72%), an overall median PFS of 9.3 months (53.3 months in patients who had achieved CR), and a median duration of response of 33.3 months [28].

It should be noted that ORR was evaluated by CT scan in the current study due to uncertainties regarding the effect of everolimus on glucose metabolism and, as a consequence, PET interpretation [6]. Due to the fact that patients with HL may have metabolically inactive residual masses posttreatment [6], it is possible that some PRs may reflect complete metabolic resolution, which may have resulted in the underestimation of ORR. Therefore, further study is necessary to evaluate this phenomenon. In addition, the 2007 recommendations for staging and assessment of treatment response in patients with and without HL were recently updated, providing new definitions of CR, PR, and progressive disease [29]. In particular, CR may be achieved in the presence of a mass that is no longer avid (i.e., one that has achieved complete metabolic response), PR may be confirmed by up to six representative nodes or extranodal lesions, and the determination of progressive disease only requires a single node [29]. Therefore, combining the improved use and interpretation of PET data along with the most recent standardized response criteria may have adjusted the assessment of response to everolimus in the current study, and thus a potentially higher ORR.

In this study, a number of patients had response durations of ≥ 1 year while being treated with everolimus on study for 2–3 years. Patients did not need to have a CR to everolimus to derive long-term positive outcomes, and a number of patients were stable on everolimus for long periods of time without disease progression. To date, there has been no evidence of a unique clinical or biologic marker that can identify patients who may derive long-term benefit with everolimus.

Everolimus was generally well tolerated, with the majority of AEs being of grade 1/2 severity and manageable without everolimus discontinuation. The need to reduce everolimus dose in approximately 60% of the patients could reflect the fact that these patients were heavily pretreated. Several of the most commonly reported AEs thought to be related to everolimus treatment (fatigue, stomatitis, rash) are consistent with those reported in previous studies of everolimus in oncology indications [30, 31]. Appropriate management strategies for preventing and treating recognized AEs associated with mTOR inhibitors as a class in both oncology and immunosuppressive settings (e.g., stomatitis, rash, pneumonitis) have been well described previously [30–32]. In the current study, all cases of pneumonitis (10.5%) were grade 1/2, with only one grade 2 event requiring recommended active management comprising adjustment or interruption of study drug dose. However, prior clinical experience of AEs associated with everolimus in patients with hematologic malignancies is not extensive, so the findings of the present trial are useful to add breadth to the current database of knowledge. In an earlier phase 2 trial in patients with lymphomas, the main grade 3/4 AEs reported in the HL subgroup were hematologic, primarily thrombocytopenia and anemia (32% each), and pulmonary, including dyspnea and pneumonia (10.5% each) [25].

In current treatment guideline recommendations for classical HL, everolimus is a subsequent systemic therapy option following second-line therapy with platinum- or gemcitabine-based chemotherapeutic regimens or brentuximab vedotin with or without platinum- or gemcitabine-based regimens [3]. Other subsequent systemic treatment options include lenalidomide, various chemotherapeutic regimens, the CPIs nivolumab (for relapsed or refractory classical HL after HSCT) and pembrolizumab (for relapsed or refractory classical HL after ≥ 3 prior lines of therapy), or second-line treatment options not previously used. Everolimus is currently in early phase testing in relapsed or refractory HL as combination therapy with lenalidomide (NCT01075321), and rituximab, dexamethasone, cytarabine, cisplatin (DHAP; NCT01453504), and an ongoing phase 2 trial is evaluating everolimus plus rituximab as maintenance therapy following high-dose chemotherapy in lymphomas (including HL; NCT01665768). In addition, we have initiated a combination trial of everolimus plus brentuximab vedotin (NCT02254239) in patients with relapsed/refractory Hodgkin lymphoma based on promising synergy in preclinical models (data not shown). Results from these everolimus combination studies are expected from late 2018, and may change the role of everolimus treatment in HL.

## Conclusions

In summary, this phase 2 trial demonstrated that everolimus monotherapy can produce a favorable ORR and median PFS in heavily pretreated patients with relapsed or refractory classical HL. The overall safety profile was consistent with that previously observed for everolimus in patients with HL and other cancers. In the setting of heavily pretreated, relapsed, or refractory HL, several novel agents (panobinostat, AFM13, nivolumab, and lenalidomide) have recently been evaluated with preliminary evidence of response [33–36]. Studies of brentuximab vedotin in a similar setting have also shown some evidence of efficacy in this population [13, 28]. In the current study, treatment with everolimus provided durable responses in a subset of patients. Therefore, the overall positive clinical outcomes for many patients in this study confirm previous findings and support further evaluation of everolimus in patients with classical HL.

## Appendix

See Table 5.

## Abbreviations

ABVD: Adriamycin^®^ (doxorubicin), bleomycin, vinblastine, and dacarbazine
AE: adverse event
AHSCT: autologous hematopoietic stem cell transplantation
BEACOPP: bleomycin, etoposide, doxorubicin, cyclophosphamide, oncovin (vincristine), procarbazine, and prednisone
CI: confidence interval
CR: complete response
CT: computed tomography
DCR: disease control rate
HL: Hodgkin lymphoma
mTOR: mammalian target of rapamycin
ORR: overall response rate
OS: overall survival
PET: positron emission tomography
PFS: progression-free survival
PI3K: phosphatidylinositol 3-kinase
PR: partial response
ULN: upper limit of normal

## References

[1] National Cancer Institute. Cancer stat facts: Hodgkin lymphoma. 2013. http://seer.cancer.gov/statfacts/html/hodg.html. Accessed 13 Jan 2017.

[2] National Cancer Institute. SEER cancer statistics review, 1975–2010. https://seer.cancer.gov/archive/csr/1975_2010. Updated 14 June 2013. Accessed 13 Jan 2017.

[3] National Comprehensive Cancer Network. Hodgkin Lymphoma (Version 3.2018). https://www.nccn.org/professionals/physician_gls/pdf/hodgkins.pdf. Accessed 9 May 2018.

[4] Kuruvilla J, Keating A, Crump M (2011). How I treat relapsed and refractory Hodgkin lymphoma. Blood.

[5] Eichenauer DA, Engert A, Dreyling M, ESMO Guidelines Working Group (2011). Hodgkin’s lymphoma: ESMO clinical practice guidelines for diagnosis, treatment and follow-up. Ann Oncol.

[6] Ramchandren R (2012). Advances in the treatment of relapsed or refractory Hodgkin’s lymphoma. Oncologist.

[7] Linch DC, Winfield D, Goldstone AH, Moir D, Hancock B, McMillan A (1993). Dose intensification with autologous bone-marrow transplantation in relapsed and resistant Hodgkin’s disease: results of a BNLI randomised trial. Lancet.

[8] Schmitz N, Pfistner B, Sextro M, Sieber M, Carella AM, Haenel M (2002). Aggressive conventional chemotherapy compared with high-dose chemotherapy with autologous haemopoietic stem-cell transplantation for relapsed chemosensitive Hodgkin’s disease: a randomised trial. Lancet.

[9] Gutierrez-Delgado F, Holmberg L, Hooper H, Petersdorf S, Press O, Maziarz R (2003). Autologous stem cell transplantation for Hodgkin’s disease: busulfan, melphalan and thiotepa compared to a radiation-based regimen. Bone Marrow Transplant.

[10] Currin ESR, Gopal AK (2012). Treatment strategies for Hodgkin lymphoma recurring following autologous hematopoietic stem cell transplantation. Korean J Hematol.

[11] Ruiz-Arguelles GJ, Lopez-Martinez B, Lopez-Ariza B (2003). Successful allogeneic stem cell transplantation with nonmyeloablative conditioning in patients with relapsed Hodgkin’s disease following autologous stem cell transplantation. Arch Med Res.

[12] Younes A, Bartlett NL, Leonard JP, Kennedy DA, Lynch CM, Sievers EL, Forero-Torres A (2010). Brentuximab vedotin (SGN-35) for relapsed CD30-positive lymphomas. N Engl J Med.

[13] Younes A, Gopal AK, Smith SE, Ansell SM, Rosenblatt JD, Savage KJ (2012). Results of a pivotal phase II study of brentuximab vedotin for patients with relapsed or refractory Hodgkin’s lymphoma. J Clin Oncol.

[14] Wang J, Yuan R, Song W, Sun J, Liu D, Li Z (2017). PD-1, PD-L1 (B7-H1) and tumor-site immune modulation therapy: the historical perspective. J Hematol Oncol.

[15] Liu B, Song Y, Liu D (2017). Recent development in clinical applications of PD-1 and PD-L1 antibodies for cancer immunotherapy. J Hematol Oncol.

[16] Bristol-Myers Squibb Company (2017). OPDIVO (nivolumab) injection, for intravenous use.

[17] Merck Sharp & Dohme Corp (2017). KEYTRUDA^®^ (pembrolizumab) for injection, for intravenous use.

[18] Jucker M, Sudel K, Horn S, Sickel M, Wegner W, Fiedler W, Feldman RA (2002). Expression of a mutated form of the p85alpha regulatory subunit of phosphatidylinositol 3-kinase in a Hodgkin’s lymphoma-derived cell line (CO). Leukemia.

[19] Dutton A, Reynolds GM, Dawson CW, Young LS, Murray PG (2005). Constitutive activation of phosphatidyl-inositide 3 kinase contributes to the survival of Hodgkin’s lymphoma cells through a mechanism involving Akt kinase and mTOR. J Pathol.

[20] Nagel S, Scherr M, Quentmeier H, Kaufmann M, Zaborski M, Drexler HG, MacLeod RAF (2005). HLXB9 activates IL6 in Hodgkin lymphoma cell lines and is regulated by PI3 K signalling involving E2F3. Leukemia.

[21] De J, Brown RE (2010). Tissue-microarray based immunohistochemical analysis of survival pathways in nodular sclerosing classical Hodgkin lymphoma as compared with non-Hodgkin’s lymphoma. Int J Clin Exp Med.

[22] Yee KW, Zeng Z, Konopleva M, Verstovsek S, Ravandi F, Ferrajoli A (2006). Phase I/II study of the mammalian target of rapamycin inhibitor everolimus (RAD001) in patients with relapsed or refractory hematologic malignancies. Clin Cancer Res.

[23] Ghobrial IM, Gertz M, Laplant B, Camoriano J, Hayman S, Lacy M (2010). Phase II trial of the oral mammalian target of rapamycin inhibitor everolimus in relapsed or refractory Waldenstrom macroglobulinemia. J Clin Oncol.

[24] Jundt F, Raetzel N, Muller C, Calkhoven CF, Kley K, Mathas S (2005). A rapamycin derivative (everolimus) controls proliferation through down-regulation of truncated CCAAT enhancer binding protein beta and NF-κB activity in Hodgkin and anaplastic large cell lymphomas. Blood.

[25] Johnston PB, Inwards DJ, Colgan JP, Laplant BR, Kabat BF, Habermann TM (2010). A phase II trial of the oral mTOR inhibitor everolimus in relapsed Hodgkin lymphoma. Am J Hematol.

[26] Simon R (1989). Optimal two-stage designs for phase II clinical trials. Control Clin Trials.

[27] Cheson BD (2007). The International Harmonization Project for response criteria in lymphoma clinical trials. Hematol Oncol Clin.

[28] Gopal AK, Chen R, Smith SE, Ansell SM, Rosenblatt JD, Savage KJ (2015). Durable remissions in a pivotal phase 2 study of brentuximab vedotin in relapsed or refractory Hodgkin lymphoma. Blood.

[29] Cheson BD, Fisher RI, Barrington SF, Cavalli F, Schwartz LH, Zucca E (2014). Recommendations for initial evaluation, staging, and response assessment of Hodgkin and non-Hodgkin lymphoma: the Lugano classification. J Clin Oncol.

[30] Porta C, Osanto S, Ravaud A, Climent MA, Vaishampayan U, White DA (2011). Management of adverse events associated with the use of everolimus in patients with advanced renal cell carcinoma. Eur J Cancer.

[31] Yardley DA (2014). Adverse event management of mTOR inhibitors during treatment of hormone receptor-positive advanced breast cancer: considerations for oncologists. Clin Breast Cancer.

[32] Kaplan B, Qazi Y, Wellen JR (2014). Strategies for the management of adverse events associated with mTOR inhibitors. Transplant Rev.

[33] Younes A, Sureda A, Ben-Yehuda D, Zinzani PL, Ong TC, Prince HM (2012). Panobinostat in patients with relapsed/refractory Hodgkin’s lymphoma after autologous stem-cell transplantation: results of a phase II study. J Clin Oncol.

[34] Rueda A, Garcia-Sanz R, Pastor M, Salar A, Labrador J, Quero-Blanco C (2015). A phase II study to evaluate lenalidomide in combination with metronomic-dose cyclophosphamide in patients with heavily pretreated classical Hodgkin lymphoma. Acta Oncol.

[35] Ansell SM, Lesokhin AM, Borrello I, Halwani A, Scott EC, Gutierrez M (2015). PD-1 blockade with nivolumab in relapsed or refractory Hodgkin’s lymphoma. N Engl J Med.

[36] Rothe A, Sasse S, Topp MS, Eichenauer DA, Hummel H, Reiners KS (2015). A phase 1 study of the bispecific anti-CD30/CD16A antibody construct AFM13 in patients with relapsed or refractory Hodgkin lymphoma. Blood.

